# Supplementary material for: Analysis of multicellular anatomical structures from spatial omics data using sosta
Source: PLoS Comput Biol. 2026 Sep 10;22(9):e1014551. doi: 10.1371/journal.pcbi.1014551 (PMC13607871; doi:10.1371/journal.pcbi.1014551)
Supplement: Supplementary file 2 [file pcbi.1014551.s002.pdf]

Response to Reviewers: “Analysis of multicellular anatomical structures from spatial omics data using *sosta*”

Editor

Thank you for submitting your manuscript and patience while awaiting peer review. In your study, a structure-based analysis framework was developed for spatial omics data. Reviewers recognized the potential importance and utility of this work. However, reviewers had raised substantive concerns that need be addressed before the manuscript can be considered further.

A few of major concerns - aggregated from reviewers comments below - are:

- The lack of quantitative benchmarking against existing tools.
- The lack of clear guidelines for optimal parameter (intensity threshold) selection, as well as insufficient evaluation of parameter sensitivity. An automated or adaptive thresholding strategy may help address this issue.

We thank the editor and reviewers for the thoughtful and constructive feedback. We have addressed the method evaluation and automated selection of parameters, amongst other suggestions by the reviewers, in our revision.

In particular, we have added a quantitative methods comparison / benchmark to compare against existing tools. Since ground truth datasets with expert annotations often exhibit subjective annotation biases (c.f., Sun et al. *2025*, https://doi.org/10.1101/2025.06.23.660861), we based our comparisons on simulations. Furthermore, this allowed us to assess performance in diverse settings, including increased background noise or differing number of cells. Our comparison revealed that our method *sosta* shows overall best-in-class performance across a range of simulation parameters. We found that GRIDGENE, a Python package that employs a similar density-based reconstruction approach as *sosta,* shows similar performance when parameters are optimally selected against the ground truth. However GRIDGENE does not offer an automated selection of parameters.

Furthermore, we clarified and refined our approach for automated parameter selection. Based on the simulations used in our methods evaluation, we demonstrate that our approach shows consistent results across a range of simulation parameters. Nevertheless, we think that manual adjustment of parameters is a valid strategy, especially given that annotation of anatomical structures has some subjectivity to it.

We have also reorganized our revised manuscript to fit the “Software” article type and hence have expanded the “Design and Implementation” section and moved the “Methods” section into our Supplementary Information file. We provide a point by point response to all reviewers comments below. In what follows, our responses are in black non-italic text; *reviewer comments are in blue italic text* and *new segments of text in the manuscript are shown in orange italic text*.

Reviewer #1

This manuscript introduces sosta, a Bioconductor package designed for "structure-based analysis" of spatial omics data. By shifting the focus from individual cells to multicellular anatomical structures using point pattern density estimation, the authors address a critical gap between classical histopathology and high-resolution molecular profiling. The utility of the framework is demonstrated through compelling case studies on colorectal cancer progression and human tonsil germinal centers. While the proposed paradigm is promising, there are several issues regarding automation, benchmarking, and biological interpretation that need to be addressed to strengthen the paper.

We thank the reviewer for the overall positive feedback on our work and helpful suggestions for improvement.

1. The current reliance on manually adjusting the density cut-off threshold introduces subjectivity and significantly hinders the method’s potential for fully automated and reproducible workflows. This dependence creates a barrier for users without prior histological knowledge or ground truth, as determining the optimal parameters for structure definition remains ambiguous. I strongly recommend implementing a more automated adaptive thresholding strategy (e.g., based on morphological stability) or, at minimum, providing comprehensive guidelines for selecting bandwidth and threshold values across diverse tissue architectures to ensure robustness.

We agree that manual dependence on parameter selection creates barriers for automated analysis. To overcome such barriers, we have implemented functions to assist in the selection of parameters. We have refined our automated selection of parameters based on kernel cross validation and selection of a density cut-off based on the intensity histogram. Furthermore, we implemented a function that gives users a visual overview of these parameter estimates; it inputs a set of images and gives the distribution of automatically-estimated values to assess the distribution of values and identify potential outliers. Our analyses have revealed that the results are consistent for a range of parameter settings.

To clarify the above, we have added to the section “Design and Implementation” the following text:

\paragraph{Parameter estimation} To estimate the cut-off threshold value c for reconstruction, we use an automated estimation of the threshold $c = \frac{m_1 + m_2}{2}$ where m_1 and m_2 are the density values at the first and second mode of the intensity distribution over all pixels in the estimated density image. In settings where the distribution is unimodal, the cut-off value lies at the peak of the distribution. We further provide auxiliary functions to facilitate manual adjustment and optimization of this cut-off value, as well as the bandwidth of the Gaussian kernel k. In particular, we provide functions to visualise density images, the intensity distributions, and to estimate the range and variability of automatically estimated parameters. Overall, we noticed that downstream results were consistent across a range of parameter settings (i.e., bandwidth of smoothing kernel and cut-off threshold), indicating robustness of our reconstruction approach. However, at an upper or lower range of values we observed substantial deviations, especially in the number of detected structures (Fig~\ref{fig:figS3}c).

Furthermore, in our systematic methods evaluation we show that our automated parameter selection strategy is robust in different scenarios and gives consistent results, please see our answer to point 2. below.

2. While the authors present structure-based analysis as a novel approach, the manuscript lacks a direct comparison with established spatial domain identification methods like CellCharter or Banksy. I recommend that the authors include a quantitative benchmark comparing sosta against these existing tools.

We first want to highlight the conceptual difference between spatial domain identification (e.g., that tools such as CellCharter or Banksy perform) with the structure-based analysis that is proposed in our manuscript. Our approach takes cell type labels, spatial domains, or other feature labels **as input**, and outputs the reconstructed anatomical structures as polygons. Based on these polygons, we then quantify geometric aspects, define distances to structure borders, and perform other structure-based analyses. Overall, we highlight across multiple scenarios that this level of resolution of the data is very relevant for various types of spatially-resolved molecular datasets. Our approach is clarified in the following passage:

Here, we introduce a new genre of data analysis, so-called structure-based analysis, whereby (computationally-extracted) anatomical structures represent the basic unit and resolution to analyse the data at (Fig~\ref{fig:fig1}c-d). As such, we complement ongoing research in cell type annotation \cite{chengBenchmarkingCellType2025} and spatial domain detection (spatial

clustering) \cite{sunBenchmarkingExpertguidedConsensus2025} since the generated cell type or domain labels are possible inputs for the reconstruction of spatial structures.

We agree that a systematic evaluation against adjacent approaches is useful since it also allows us to test our automated parameter selection strategy. We based our comparison on simulations and compared against four methods that also take celltype or other labels as input and output polygons (CellCharter, GRIDGENE, imcRtools, SPIAT). We show that *sosta* shows overall best-in-class performance across a range of parameters. We found that GRIDGENE, a method that employs a similar density-based reconstruction approach in Python shows similar performance under optimal parameter selection choices. However, it requires manual selection of all parameters. Below is a figure showing the performance of the evaluated methods (corresponds to Suppl. Fig 2c).

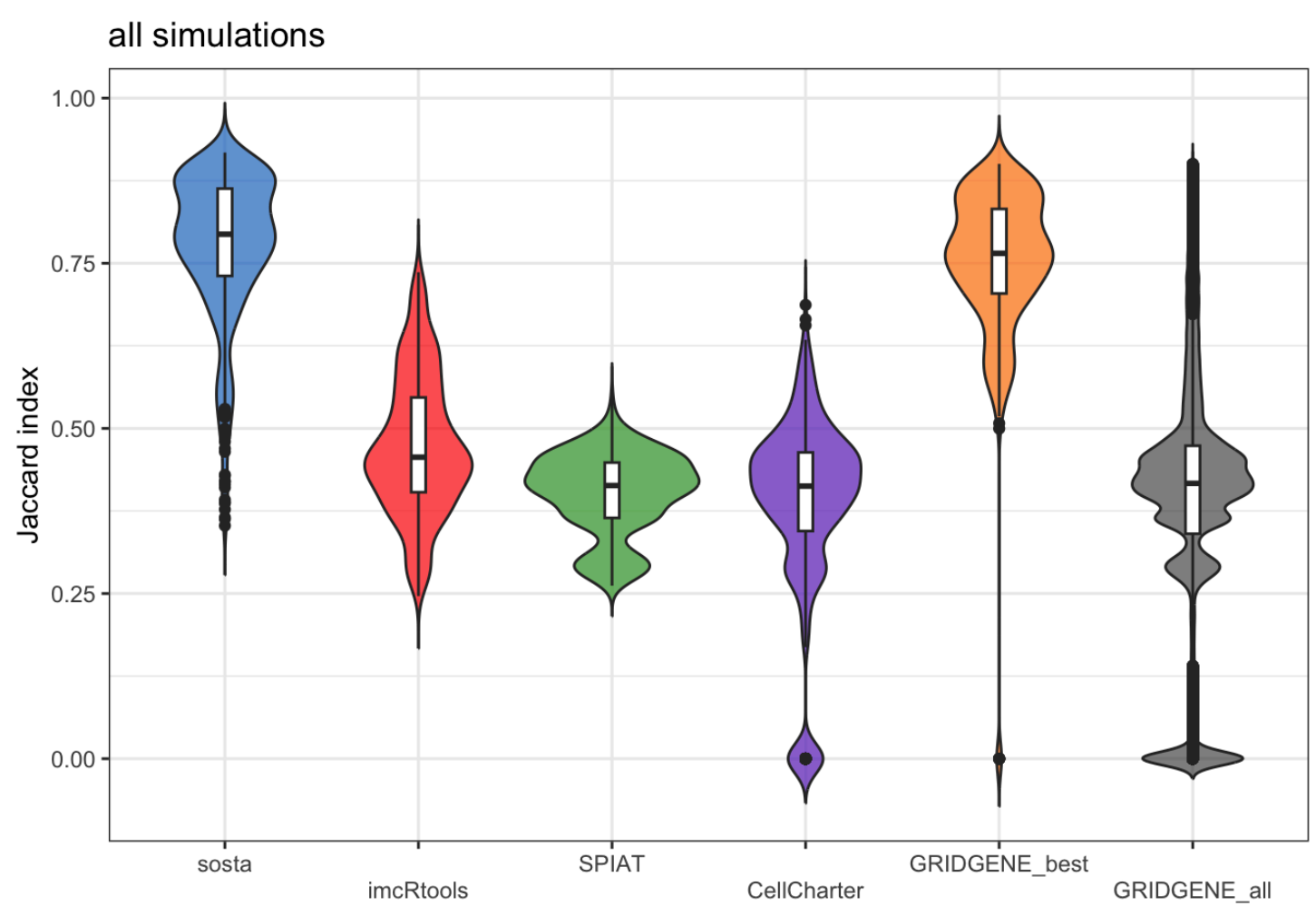

We have added the paragraph "Methods comparison" to our manuscript, added Suppl Figure 2 and described the full approach in the Supplementary Information.

3. The authors observe that 'Fibre Width' increases with pathological progression and correlates with pseudo-time. However, the biological substance underlying these geometric metrics needs to be more explicitly defined. Specifically, the authors should clarify whether the increase in Fibre Width serves as a proxy for biological processes such as epithelial hyperplasia or loss of cell polarity, rather than presenting it solely as a mathematical observation.

Indeed, the metric "fibre width" was chosen as a proxy for increase in epithelial crypt thickness which is progressive from normal to hypertrophy and hyperplasia. We have clarified the biological interpretation of an increase in fibre width in the text as follows.

Fibre width increased along the pathological progression from HC to TVA to CRC, both at the structure and cell type level (Fig~\ref{fig:fig2}d-e). As such, this metric reflects the gradual morphological changes related to increase in crypt thickness. In healthy tissue, epithelial cells are lining the crypt; in hypertrophy and hyperplasia, there is an increase in size and number of epithelial cells, respectively. Next, dysplasia denotes a known premalignant state in which epithelial cells lose differentiation, present abnormal size and shape and lack polarization followed by carcinoma \cite{flemingColorectalCarcinomaPathologic2012, galuppiniHistomorphologicalMolecularLandscape2021}.

4. In the CRC analysis, the authors identify 'transition crypts' using algorithmic thresholds. To validate the reliability of this strategy, I recommend providing a confusion matrix (along with Precision and Recall metrics) comparing the algorithmically detected crypts against expert pathologist manual annotations.

We have not added a confusion matrix since we could not validate all *de novo* identified transitions crypts. However, we have added a statement in the text about the number of transition crypts that we recalled based on the manual annotation. While we only recovered one of seven crypts, we still think that this approach can be interesting for exploratory analyses since we show that one *de novo* identified crypt also showed characteristic morphological changes in the histopathology image (Suppl. Fig 1g). To illustrate the potential false positives during this approach, we have added an example of a falsely identified transition crypt (Suppl. Fig 1h-i) and changed the main text to:

By filtering based on the frequency of cell types adjacent to the reconstructed crypts, we recovered one of seven manually annotated transition crypts and identified potentially \textit{de novo} transition crypts. Although this approach failed to detect several manually annotated crypts, it revealed additional candidate crypts that also showed transition patterns in the histopathology image (\hyperref[sec:supp-methods]{Suppl. Methods}, Fig~\ref{fig:fig2}i-j, Suppl. Fig~\ref{fig:figS3}d-h).

Reviewer #2

The manuscript introduces sosta, a structure-based analysis framework and an R/Bioconductor package designed for spatial omics data. The authors correctly identify a critical gap in the field: while most current methods prioritize single-cell spatial distributions, biological functions are often driven by higher-order multicellular anatomical structures. My comments are as follows:

We appreciate the reviewer’s constructive feedback.

1 While the results presented are quite compelling, the manuscript does not include a comparative analysis with any existing methodologies. Consequently, it is difficult to assess the actual impact and standing of the proposed method within the field.

We have expanded our analysis to include a comparison with existing methodologies. Compared to four other methods that take cell coordinates as input and output polygons that can be used for downstream structure-based analysis, *sosta* shows best-in-class performance. We refer to the response to Reviewer #1, point 2. for more details on the methods comparison.

2 For instance, in the area of cell-type annotation, a significant number of methods have already been established; these warrant a thorough discussion and comparison.

We want to stress that *sosta* represents a conceptually different approach to cell type annotation, and to spatial domain detection. The input of our approach are cells with labels and the output are polygons that can be used for structure-based analysis. We have clarified the scope and limitation of our approach in the main text in the Introduction. See the response to Reviewer #1, point 2. for a more detailed explanation.

3 Furthermore, the authors should contextualize their work within the broader landscape of spatial omics computational research. Specifically, it would be beneficial to discuss and compare the proposed approach with relevant methods such as STAGATE, SpaMask, mclSTExp, SpaBatch, SpaCross, and SpaDAMA.

We thank the reviewer for this viewpoint. In the response to Reviewer #1, point 2, we have tried to better place how *sosta* fits into the landscape of spatial omics data analysis research. In particular, we have now better expressed how *sosta* is different to spatial domain detection (e.g., STAGATE and SpaMask in the list above). However, some of the tools mentioned above do not seem to truly fit the context of *sosta* and are not mentioned in the revision. For example, SpaDAMA is a tool for lower-dimensional spot-based spatial transcriptomics data together with single cell RNA-seq data; SpaBatch and SpaCross are about aligned multiple slices of tissue; mclSTExp is about predicting expression from H&E images.

Below is our modified text:

Here, we introduce a new genre of data analysis, so-called structure-based analysis, whereby (computationally-extracted) anatomical structures represent the basic unit and resolution to analyse the data at (Fig~\ref{fig:fig1}c-d). As such, we complement ongoing research in cell type annotation \cite{chengBenchmarkingCellType2025} and spatial domain detection (spatial

clustering) \cite{sunBenchmarkingExpertguidedConsensus2025} since the generated cell type or domain labels are possible inputs for the reconstruction of spatial structures.

And the following paragraph in the "Discussion" section.

While our framework offers methods to reconstruct, quantify and compare anatomical structures ranging from single to multi-sample multi-condition setups, we acknowledge that there exists a plethora of methods that can be applied in each step of structure-based analysis. First, suitable targets for reconstruction have to be identified. These could range from marker expression, cell type labels, pathway signatures to spatial domains. Second, a suitable reconstruction method and parameters have to be chosen. Third, the range of metrics for downstream analyses have to be identified. For example, a range of methods exists to reconstruct and segment (multi-)cellular structures, the space of possible metrics that quantify anatomical structures is vast and different strategies to detect gradients exist \cite{nicolIdentifyingSpatiallyVariable2024}. Nonetheless, our flexible and extensible framework can accommodate future developments in this space.
