# Supplementary material for: Analysis of multicellular anatomical structures from spatial omics data using sosta
Source: PLoS Comput Biol. 2026 Sep 10;22(9):e1014551. doi: 10.1371/journal.pcbi.1014551 (PMC13607871; doi:10.1371/journal.pcbi.1014551)
Supplement: Peer review history

# Peer review: Decision Letter 0

**DOI:** 10.1371/journal.pcbi.1014551.r001

**Authors:** Ilya Ioshikhes, Boyang Ji

11 Feb 2026

Analysis of anatomical multi-cellular structures from spatial omics data using sosta

PLOS Computational Biology

Dear Dr. Robinson,

Thank you for submitting your manuscript to PLOS Computational Biology. After careful consideration, we feel that it has merit but does not fully meet PLOS Computational Biology's publication criteria as it currently stands. Therefore, we invite you to submit a revised version of the manuscript that addresses the points raised during the review process.

Please submit your revised manuscript by Apr 13 2026 11:59PM. If you will need more time than this to complete your revisions, please reply to this message or contact the journal office at ploscompbiol@plos.org. When you're ready to submit your revision, log on to https://www.editorialmanager.com/pcompbiol/ and select the 'Submissions Needing Revision' folder to locate your manuscript file.

Please include the following items when submitting your revised manuscript:

* A letter that responds to each point raised by the editor and reviewer(s). You should upload this letter as a separate file labeled 'Response to Reviewers'. This file does not need to include responses to formatting updates and technical items listed in the 'Journal Requirements' section below.

* A marked-up copy of your manuscript that highlights changes made to the original version. You should upload this as a separate file labeled 'Revised Manuscript with Track Changes'.

* An unmarked version of your revised paper without tracked changes. You should upload this as a separate file labeled 'Manuscript'.

If you would like to make changes to your financial disclosure, competing interests statement, or data availability statement, please make these updates within the submission form at the time of resubmission. Guidelines for resubmitting your figure files are available below the reviewer comments at the end of this letter

We look forward to receiving your revised manuscript.

Kind regards,

Boyang Ji, Ph.D.

Academic Editor

PLOS Computational Biology

Ilya Ioshikhes

Section Editor

PLOS Computational Biology

**Additional Editor Comments:**

Dear Prof. Robinson, and co-authors,

Thank you for submitting your manuscript and patience while awaiting peer review. In your study, a structure-based analysis framework was developed for spatial omics data. Reviewers recognized the potential importance and utility of this work. However, reviewers had raised substantive concerns that need be addressed before the manuscript can be considered further.

A few of major concerns - aggregated from reviewers comments below - are:

- The lack of quantitative benchmarking against existing tools.

- The lack of clear guidelines for optimal parameter (intensity threshold) selection, as well as insufficient evaluation of parameter sensitivity. An automated or adaptive thresholding strategy may help address this issue.

**Journal Requirements:**

If the reviewer comments include a recommendation to cite specific previously published works, please review and evaluate these publications to determine whether they are relevant and should be cited. There is no requirement to cite these works unless the editor has indicated otherwise.

1) We ask that a manuscript source file is provided at Revision. Please upload your manuscript file as a .doc, .docx, .rtf or .tex. If you are providing a .tex file, please upload it under the item type u2018LaTeX Source Fileu2019 and leave your .pdf version as the item type u2018Manuscriptu2019.

2) Your manuscript is missing the following sections: Design and Implementation, and Availability and Future Directions. Please ensure that your article adheres to the standard Software article layout and order of Abstract, Introduction, Design and Implementation, Results, and Availability and Future Directions. For details on what each section should contain, see our Software article guidelines:

https://journals.plos.org/ploscompbiol/s/submission-guidelines#loc-software-submissions

3) Please upload all main figures as separate Figure files in .tif or .eps format. For more information about how to convert and format your figure files please see our guidelines:

https://journals.plos.org/ploscompbiol/s/figures

4) We have noticed that you have uploaded Supporting Information files, but you have not included a list of legends. Please add a full list of legends for your Supporting Information files after the references list.

5) Some material included in your submission may be copyrighted. According to PLOSu2019s copyright policy, authors who use figures or other material (e.g., graphics, clipart, maps) from another author or copyright holder must demonstrate or obtain permission to publish this material under the Creative Commons Attribution 4.0 International (CC BY 4.0) License used by PLOS journals. Please closely review the details of PLOSu2019s copyright requirements here: PLOS Licenses and Copyright. If you need to request permissions from a copyright holder, you may use PLOS's Copyright Content Permission form.

Please respond directly to this email and provide any known details concerning your material's license terms and permissions required for reuse, even if you have not yet obtained copyright permissions or are unsure of your material's copyright compatibility. Once you have responded and addressed all other outstanding technical requirements, you may resubmit your manuscript within Editorial Manager.

Potential Copyright Issues:

i) Figures 1A, and 1c. Please confirm whether you drew the images / clip-art within the figure panels by hand. If you did not draw the images, please provide (a) a link to the source of the images or icons and their license / terms of use; or (b) written permission from the copyright holder to publish the images or icons under our CC BY 4.0 license. Alternatively, you may replace the images with open source alternatives. See these open source resources you may use to replace images / clip-art:

- https://commons.wikimedia.org

- https://openclipart.org/.

6) Please amend your detailed Financial Disclosure statement. This is published with the article. It must therefore be completed in full sentences and contain the exact wording you wish to be published.

1) State the initials, alongside each funding source, of each author to receive each grant. For example: "This work was supported by the National Institutes of Health (####### to AM; ###### to CJ) and the National Science Foundation (###### to AM)."

2) State what role the funders took in the study. If the funders had no role in your study, please state: "The funders had no role in study design, data collection and analysis, decision to publish, or preparation of the manuscript."

3) If any authors received a salary from any of your funders, please state which authors and which funders..

If you did not receive any funding for this study, please simply state: u201cThe authors received no specific funding for this work.u201d

**Reviewers' comments:**

Reviewer's Responses to Questions

**Comments to the Authors:**

**Please note here if the review is uploaded as an attachment.**

Reviewer #1: This manuscript introduces sosta, a Bioconductor package designed for "structure-based analysis" of spatial omics data. By shifting the focus from individual cells to multicellular anatomical structures using point pattern density estimation, the authors address a critical gap between classical histopathology and high-resolution molecular profiling. The utility of the framework is demonstrated through compelling case studies on colorectal cancer progression and human tonsil germinal centers. While the proposed paradigm is promising, there are several issues regarding automation, benchmarking, and biological interpretation that need to be addressed to strengthen the paper.

1. The current reliance on manually adjusting the density cut-off threshold introduces subjectivity and significantly hinders the method’s potential for fully automated and reproducible workflows. This dependence creates a barrier for users without prior histological knowledge or ground truth, as determining the optimal parameters for structure definition remains ambiguous. I strongly recommend implementing a more automated adaptive thresholding strategy (e.g., based on morphological stability) or, at minimum, providing comprehensive guidelines for selecting bandwidth and threshold values across diverse tissue architectures to ensure robustness.

2. While the authors present structure-based analysis as a novel approach, the manuscript lacks a direct comparison with established spatial domain identification methods like CellCharter or Banksy. I recommend that the authors include a quantitative benchmark comparing sosta against these existing tools.

3. The authors observe that 'Fibre Width' increases with pathological progression and correlates with pseudo-time. However, the biological substance underlying these geometric metrics needs to be more explicitly defined. Specifically, the authors should clarify whether the increase in Fibre Width serves as a proxy for biological processes such as epithelial hyperplasia or loss of cell polarity, rather than presenting it solely as a mathematical observation.

4. In the CRC analysis, the authors identify 'transition crypts' using algorithmic thresholds. To validate the reliability of this strategy, I recommend providing a confusion matrix (along with Precision and Recall metrics) comparing the algorithmically detected crypts against expert pathologist manual annotations.

Reviewer #2: The manuscript introduces sosta, a structure-based analysis framework and an R/Bioconductor package designed for spatial omics data. The authors correctly identify a critical gap in the field: while most current methods prioritize single-cell spatial distributions, biological functions are often driven by higher-order multicellular anatomical structures. My comments are as follows:

1 While the results presented are quite compelling, the manuscript does not include a comparative analysis with any existing methodologies. Consequently, it is difficult to assess the actual impact and standing of the proposed method within the field.

2 For instance, in the area of cell-type annotation, a significant number of methods have already been established; these warrant a thorough discussion and comparison.

3 Furthermore, the authors should contextualize their work within the broader landscape of spatial omics computational research. Specifically, it would be beneficial to discuss and compare the proposed approach with relevant methods such as STAGATE, SpaMask, mclSTExp, SpaBatch, SpaCross, and SpaDAMA.

**********

**Have the authors made all data and (if applicable) computational code underlying the findings in their manuscript fully available?**

The PLOS Data policy requires authors to make all data and code underlying the findings described in their manuscript fully available without restriction, with rare exception (please refer to the Data Availability Statement in the manuscript PDF file). The data and code should be provided as part of the manuscript or its supporting information, or deposited to a public repository. For example, in addition to summary statistics, the data points behind means, medians and variance measures should be available. If there are restrictions on publicly sharing data or code —e.g. participant privacy or use of data from a third party—those must be specified.

Reviewer #1: None

Reviewer #2: Yes

**********

PLOS authors have the option to publish the peer review history of their article (what does this mean?). If published, this will include your full peer review and any attached files.

If you choose “no”, your identity will remain anonymous but your review may still be made public.

**Do you want your identity to be public for this peer review?**  For information about this choice, including consent withdrawal, please see our Privacy Policy.

Reviewer #1: No

Reviewer #2: No

**Figure resubmission:**

**Reproducibility:**

?>

# Author comment: Author response to Decision Letter 1

**DOI:** 10.1371/journal.pcbi.1014551.r002

9 Apr 2026

Attachment Submitted filename: sosta_revision-responsetoreviewers.pdf

# Peer review: Decision Letter 1

**DOI:** 10.1371/journal.pcbi.1014551.r003

**Authors:** Ilya Ioshikhes, Boyang Ji

9 Jul 2026

Dear Prof Robinson,

We are pleased to inform you that your manuscript 'Analysis of multicellular anatomical structures from spatial omics data using sosta' has been provisionally accepted for publication in PLOS Computational Biology.

Before your manuscript can be formally accepted you will need to complete some formatting changes, which you will receive in a follow up email. A member of our team will be in touch with a set of requests.

Please note that your manuscript will not be scheduled for publication until you have made the required changes, so a swift response is appreciated.

IMPORTANT: The editorial review process is now complete. PLOS will only permit corrections to spelling, formatting or significant scientific errors from this point onwards. Requests for major changes, or any which affect the scientific understanding of your work, will cause delays to the publication date of your manuscript.

Should you, your institution's press office or the journal office choose to press release your paper, you will automatically be opted out of early publication. We ask that you notify us now if you or your institution is planning to press release the article. All press must be co-ordinated with PLOS.

Thank you again for supporting Open Access publishing; we are looking forward to publishing your work in PLOS Computational Biology.

Best regards,

Chongzhi Zang

Academic Editor

PLOS Computational Biology

Ilya Ioshikhes

Section Editor

PLOS Computational Biology

***********************************************************

I apologize for the delay in the editorial process for this manuscript, as I was only recently assigned as the editor. One of the reviewers has been non-responsive; however, after reviewing the manuscript, the previous reviews, and the authors' response to the reviewers' comments, I am convinced that the reviewers' concerns have been well addressed. I am therefore pleased to accept the revised manuscript for publication in PLOS Computational Biology.

Reviewer's Responses to Questions

**Comments to the Authors:**

**Please note here if the review is uploaded as an attachment.**

Reviewer #2: In this version of the manuscript, the authors have addressed my questions satisfactorily, and I have no further comments.

**********

**Have the authors made all data and (if applicable) computational code underlying the findings in their manuscript fully available?**

The PLOS Data policy requires authors to make all data and code underlying the findings described in their manuscript fully available without restriction, with rare exception (please refer to the Data Availability Statement in the manuscript PDF file). The data and code should be provided as part of the manuscript or its supporting information, or deposited to a public repository. For example, in addition to summary statistics, the data points behind means, medians and variance measures should be available. If there are restrictions on publicly sharing data or code —e.g. participant privacy or use of data from a third party—those must be specified.

Reviewer #2: Yes

**********

PLOS authors have the option to publish the peer review history of their article (what does this mean?). If published, this will include your full peer review and any attached files.

If you choose “no”, your identity will remain anonymous but your review may still be made public.

**Do you want your identity to be public for this peer review?**  For information about this choice, including consent withdrawal, please see our Privacy Policy.

Reviewer #2: No

# Editor report: Acceptance letter

**DOI:** 10.1371/journal.pcbi.1014551.r004

**Authors:** Ilya Ioshikhes, Boyang Ji

PCOMPBIOL-D-26-00028R1

Analysis of multicellular anatomical structures from spatial omics data using sosta

Dear Dr Robinson,

I am pleased to inform you that your manuscript has been formally accepted for publication in PLOS Computational Biology. Your manuscript is now with our production department and you will be notified of the publication date in due course.

The corresponding author will soon be receiving a typeset proof for review, to ensure errors have not been introduced during production. Please review the PDF proof of your manuscript carefully, as this is the last chance to correct any errors. Please note that major changes, or those which affect the scientific understanding of the work, will likely cause delays to the publication date of your manuscript.

Soon after your final files are uploaded, unless you have opted out, the early version of your manuscript will be published online. The date of the early version will be your article's publication date. The final article will be published to the same URL, and all versions of the paper will be accessible to readers.

For Research, Software, and Methods articles, you will receive an invoice from PLOS for your publication fee after your manuscript has reached the completed accept phase. If you receive an email requesting payment before acceptance or for any other service, this may be a phishing scheme. Learn how to identify phishing emails and protect your accounts at https://explore.plos.org/phishing.

Thank you again for supporting PLOS Computational Biology and open-access publishing. We are looking forward to publishing your work!

With kind regards,

Sharmila Kamatchi

PLOS Computational Biology | Carlyle House, Carlyle Road, Cambridge CB4 3DN | United Kingdom ploscompbiol@plos.org | Phone +44 (0) 1223-442824 | ploscompbiol.org | @PLOSCompBiol
